# Primary Mandibular Condyle Xanthoma: Case Report and Literature Review

**DOI:** 10.3390/reports6010006

**Published:** 2023-02-15

**Authors:** Sara Negrello, Arrigo Pellacani, Mattia Di Bartolomeo, Giuseppe Pollastri, Alexandre Anesi

**Affiliations:** 1 Cranio-Maxillo-Facial Surgery Unit, University Hospital of Modena, 41124 Modena, Italy; 2 Unit of Dentistry and Maxillo-Facial Surgery, Surgery, Dentistry, Maternity and Infant Department, University of Verona, P.le L.A. Scuro 10, 37134 Verona, Italy; 3 Department of Medical and Surgical Sciences for Children & Adults, Cranio-Maxillo-Facial Surgery, University of Modena and Reggio Emilia, Largo del Pozzo 71, 41124 Modena, Italy

**Keywords:** xanthoma, mandibular condyle, temporomandibular joint, mandibular lesions, cone-beam computed tomography

## Abstract

Bone xanthoma is a rare benign primary bone lesion histologically characterized by sheets of foamy cells which are macrophages with a cytoplasm filled with droplets of fat. It is usually associated with endocrine or metabolic diseases, in the absence of which the lesion is called primary xanthoma. Because of the lack of pathognomonic radiologic and clinical features, they require a differential diagnosis with a broad spectrum of lesions with a varying degree of malignant potential. We describe a case of primary mandibular xanthoma of a 16-year-old girl without typical cutaneous manifestations and alterations in lipid values. The temporomandibular joint involvement at the mandibular condyle is peculiar in the case described here. We present even a qualitative systematic review of the literature on primary xanthoma of the jaws in others to draw up treatment guidelines.

## 1. Introduction

Xanthoma (from the Greek ‘xhantÓs’ that means yellow) is a non-neoplastic granulomatous lesion commonly occurring in soft tissue such as skin, subcutaneous tissue, or tendon sheaths, and is caused by abnormal deposition of cholesterol and the resulting inflammatory cell infiltration [1].

It commonly represents a benign secondary manifestation of systemic diseases that affect the lipid or glucose metabolism, such as dyslipidaemias or diabetes.

Intraosseous xanthomas are uncommon, especially the so-called primary xanthomas not related to systemic alteration [2,3]. The most frequent bone locations are the hand and the diaphysis of long bones, especially the tibia, while in the jaws it is extremely rare, and seems to be always primary and occurs quite exclusively in mandible.

Their diagnosis can be challenging because they are often incidentally detected on panoramic radiographs as well as on dental intraoral radiographs and, furthermore, because their radiological presentation may be non-specific.

It is important to know their characteristics and take them into account during differential diagnosis of mandibular bone lesions.

We present a case of mandibular xanthoma and a review of the literature.

## 2. Case Presentation Section and Review of the Literature

### 2.1. Case Report

**Figure 1:**
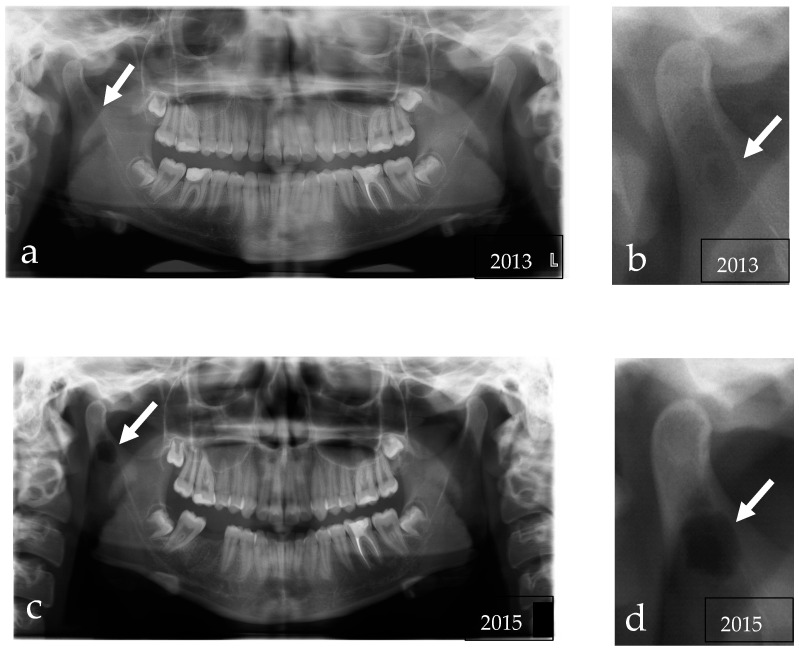
(**a**) The Rx Orthopantomography made in 2013 showing a diffuse, unilocular, and radiolucent lesion, with regular margins, located within condylar process; (**b**) Close view of the lesion; (**c**) The panoramic radiograph made in 2015 showing a small unilocular osteolytic lesion at the base of the right condylar process; (**d**) Close view of the lesion in the panoramic radiograph.

In May 2016, a 16-year-old girl was referred to Cranio–Maxillo-Facial Unit of the University Hospital of Modena for the incidental detection of a mandibular lesion in an orthopantomography performed for orthodontic care. The panoramic radiograph showed an osteolytic lesion with well-defined contours in the area of the right mandibular condyle (Figure 1c,d).

The patient did not report any symptoms, no facial paresthesia was observed, and she did not complain about pain. The examination was noncontributory: there was no swelling, the face was symmetric, oral mucous presented normal color and consistence without visible lesions, there were no carious teeth, periodontal pockets, or other visible dental disease, and there was no evidence of regional lymph node enlargement. The patient presented a second-class occlusion, and there was a deviation of the mandibular midline toward the right.

The personal history reported asymptomatic hyperprolactinemia of 30 ng/mL (the magnetic resonance imaging (MRI) did not detect pituitary disorders) and seborrheic dermatitis, but the patient denied previous local trauma and the family history was negative for neoplastic diseases or metabolic disorders. The girl did not smoke and did not drink alcohol.

Routine laboratory tests, including measurement of serum calcium (4.6 mEq/L), phosphorus (4 mg/dL), alkaline phosphatase levels (50 IU/L), glycaemia (80 mg/dL), and cholesterol (LDL 90 md/dL) were within normal limits and so we excluded metabolic abnormalities.

**Figure 2:**
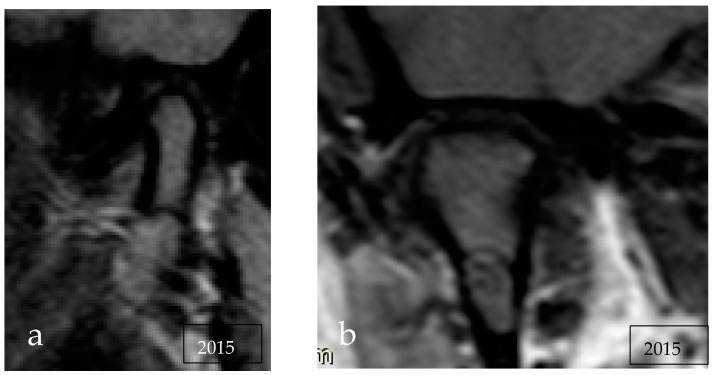
MRI scans showing an intraosseous lesion isointense to bone marrow and with a hypointense rib: (**a**) sagittal projection, (**b**) coronal projection.

We reviewed her previous radiological images. In 2013, a panoramic radiograph already showed a small unilocular osteolytic lesion at the base of the right condylar process (Figure 1a,b) but it had been reported as non-pathological. Then, to investigate a click of the left temporomandibular joint, the patient performed a dynamic MRI that confirmed the presence of an intraosseous lesion at the level of the right mandibular condyle, isointense to bone marrow on T1 and hyperintense on T2 and with a hypointense rib, suggesting content rich of fat and fibrous capsule (Figure 2).

**Figure 3:**
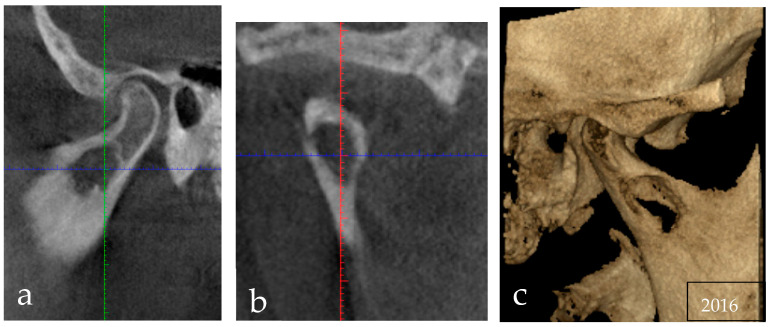
Preoperative cone-beam computed tomography (CBCT) with multiplanar reconstructions showing a unilocular osteolytic lesion, with relatively well-defined but irregular borders and sclerotic margin, extending into the right mandibular condyle; there was not, however, cortical expansion or deformation of the condylar process: (**a**) sagittal view, (**b**) coronal view, (**c**) three-dimensional reconstruction. Images were obtained at 120 kVp e 3.8 mA and reconstructed using voxel size of 0.3. The green line is the indicator line of the coronal slice in the sagittal sections, the red line is the indicator line of the sagittal slice in the coronal sections, and the blue lines are the indicator lines of the axial slices in the sagittal (**a**) and coronal (**b**) sections.

Since the patient came to visit three years after this finding, with only a new OPT made in 2015, it was necessary to perform an additional X-ray examination to check the current size and the exact position of the lesion at the time of the treatment planning. To avoid further radiation of a traditional computed tomography (CT) scan, a cone-beam CT was asked for that revealed a mandibular osteolytic unilocular lesion, with relatively well-defined but irregular borders and sclerotic margin, extending into the right mandibular condyle; however, cortical expansion or deformation of the condylar process were not present. The lesion was not related to the teeth (Figure 3).

The initial differential diagnosis included a lot of mandibular diseases that appear as a well-defined, radiolucent lesion, for example: odontogenic cysts or tumors, vascular malformation, traumatic bone cyst, and central giant cell tumor.

Based on the clinical examination and the laboratory values, an inflammatory process could be eliminated from the differential diagnosis. Moreover, the defined limit and the lack of cortical erosion did not suggest a primary or metastatic malignant neoplasm.

In view of the probable benign nature of the lesion and the importance of preserving the joint function, we performed an excisional biopsy followed by a curettage of the bone cavity. The patient was treated in 2016, before the COVID-19 pandemic, when lesions with very slow growth patterns were not postponed [4].

**Figure 4:**
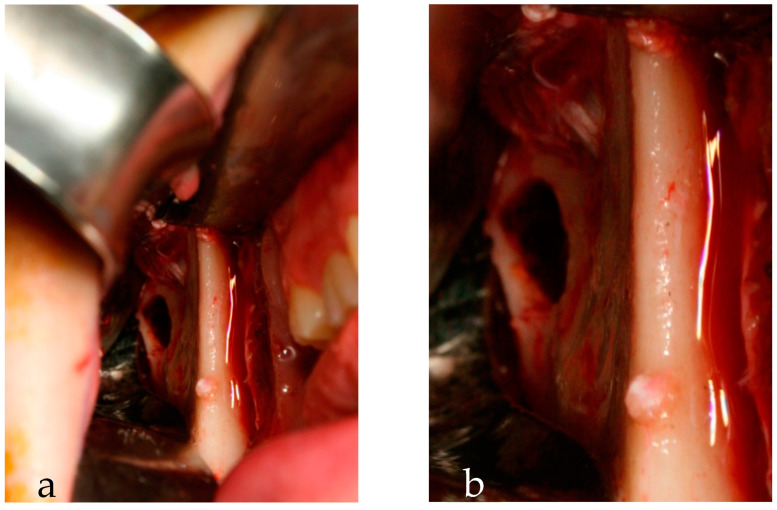
Intra-operative pictures. (**a**) Mandibular bone defect after surgical excision, and (**b**) a particular view.

Under general anesthesia by nasotracheal intubation, a right intraoral incision was made in the mucosa of the lower vestibule to expose the cortical bone of the mandibular branch and the condyle, which appeared intact. With a bur, we consumed the bone to expose the thin wall of the lesion filled with a yellowish-gray amorphous tissue that was fully removed and curetted. We filled the bone cavity with sponges of fibrin and sutured the mucosa wound with Vicryl thread 4.0. (Figure 4).

Histological examination revealed aggregates of foamy histiocytes in scarce fibrous tissue with isolated lymphocytes and plasma cells.

Immunohistochemically, the foam cells presented positivity for CD68, negativity for cytokeratin (mAb MNF116, CK7) CD34, and negativity for S100 protein and CD1a (excluding Langerhans cells).

The postoperative hospital stay was uneventful.

**Figure 5:**
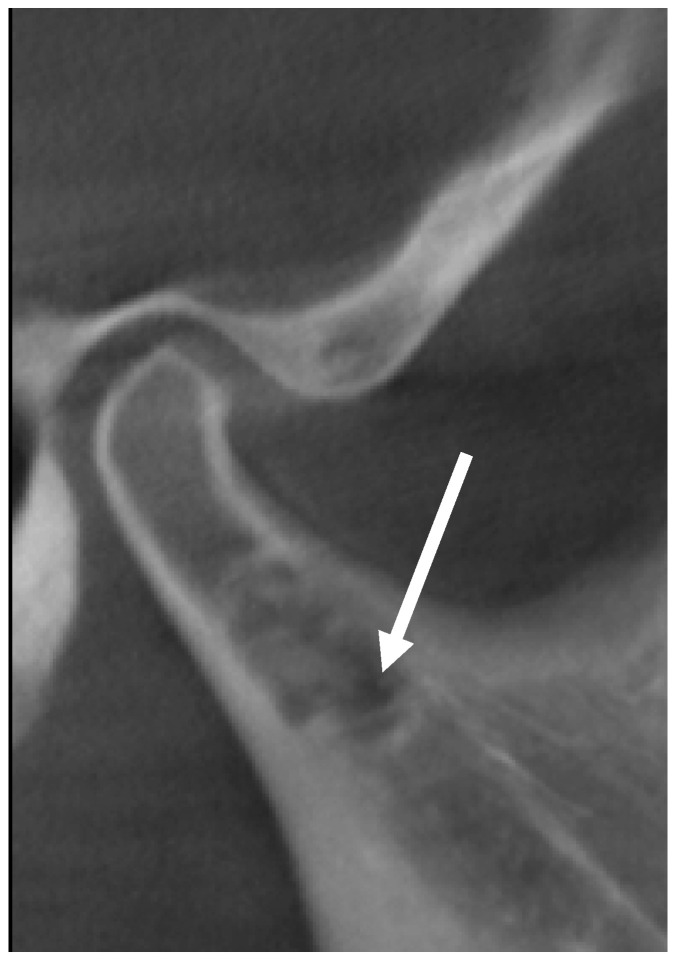
Postoperative cone-beam CT performed 6 months after surgery. Bone regeneration is evident, and only a small central area of lower bone density is not detectable (white arrow).

In October 2016, a new CBCT demonstrated a good ossification of the surgical site, with only a small residual central area of lower bone density (Figure 5).

No signs or symptoms of recurrence were observed in the 6 years of clinical and radiological follow up. The patient conserved a normal function of the temporomandibular joint.

### 2.2. Literature Review

A systematic review in accordance with the Preferred Reporting Items for Systematic reviews and Meta-Analyses (PRISMA) statement for reporting systematic reviews was conducted [5].

#### 2.2.1. Focus Questions

The focus questions were:

- What is the pathogenesis of primary bone xanthoma of the jaw?
- Is surgery the gold standard in the treatment of bone xanthoma?
- Can we preserve teeth or other noble structure?
- Is there a more suggestive radiological aspect?
- Is biopsy always mandatory?
- Is follow up necessary?

#### 2.2.2. Search Strategy

A literature search was performed in PubMed, Scopus, and Web of Science for all articles within bone xanthomas of the jaws, published as far as June 2022, using the search strings: (xanthoma OR xanthomas OR xanthomatosis OR xanthomatous) AND (mandible OR maxilla OR jaw OR jaws).

#### 2.2.3. Selection Criteria

The following inclusion criteria were adopted based on the PICOS criteria [5]: (P) Patients: patients of all ages and sexes with a primary xamthoma of the jaws; (I) Intervention: patients with bone xanthoma who underwent surgical procedure; (C) Comparator: not applicable; (O) Outcomes: incidence of relapse and complications; and (S) Study design: clinical human studies, including randomized controlled trials, controlled clinical trials, retrospective cohort studies, and case reports of case series with the aim of investigating functional results and efficacy of the surgical treatment.

The research was limited to papers published in English.

#### 2.2.4. Exclusion Criteria

The following exclusion criteria were applied:

(1) patients without a bone xanthoma, (2) patients with systemic disease (3), patients with visceral or skin lesions, (4) patients not surgical treated, (5) studies not in English, (6) animal or in vitro studies, and (7) studies published more than once reporting the same patients.

#### 2.2.5. Data Extraction

Data were extracted independently by two authors (S.N. and A.P.) using the same formula. The following information was extracted from each study: first author and year of publication, study design, number and gender of patients, age at diagnosis, location and size of lesion, clinical and radiological features, type of treatment, histological features, relapse and complications, and follow-up period (Table 1).

#### 2.2.6. Quality Assessment of Included Studies and Bias

The selected studies were almost all case reports or case series with a limited number of patients and without a control group. Their authors only described the clinical and radiological aspect of the lesions and surgical procedure efficacy. For all these considerations, we could only conduct a qualitative systematic review.

#### 2.2.7. Results

A total of 122 articles were identified utilizing the initial query on PubMed, Scopus, and Web of Science. After removing the duplicates, we obtained 62 articles. Of these, applying the selection criteria, 42 were excluded based on the title, and 1 article was excluded after reading the abstract.

**Figure 6:**
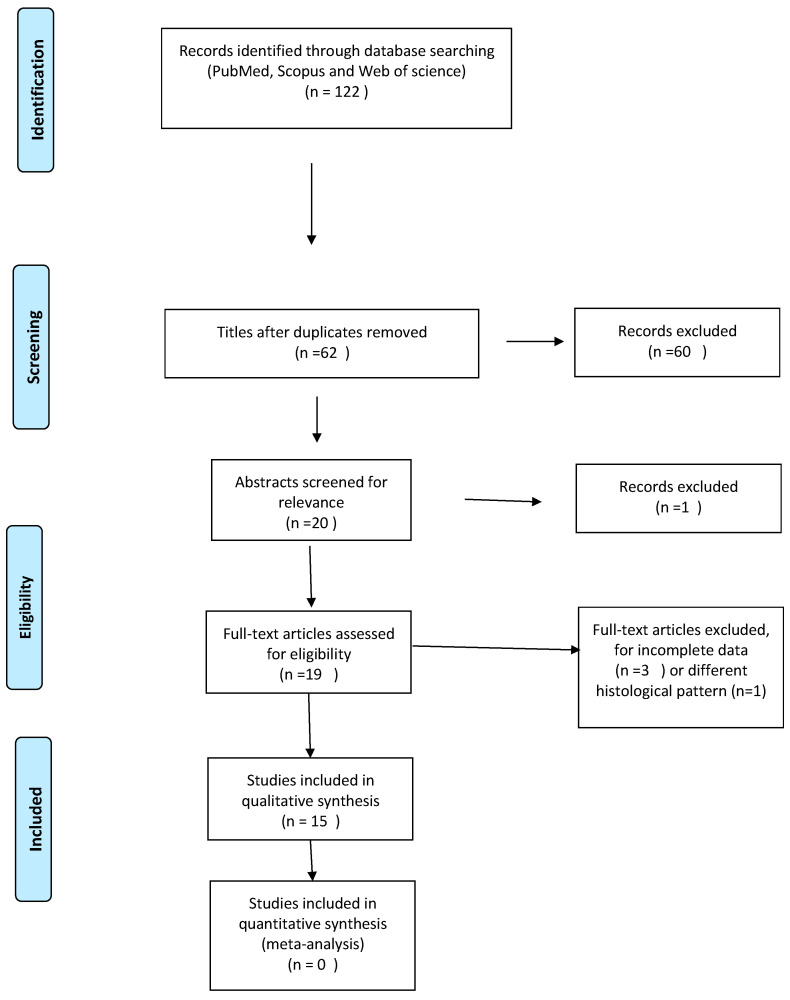
Flow chart articles exclusion.

After reading the full texts, 15 articles were identified and used for the analysis, 3 were excluded because of incomplete data, and another 1 was excluded because of a case of uncontrolled diabetes [6] (Figure 6) (Table S1).

A total of 43 cases of primary intraosseous xanthoma of the jaws were selected, 19 cases in males and 24 cases in females. The mean age of the patients was 29 (range from 11 to 72 years) (Table 1).

Forty cases occurred in the mandible and three in the maxillary bone, twenty-two in the posterior left mandible and fourteen in the right, and only four in anterior mandible.

The lesions described were mostly small in size, between 2 and 3 cm, except for one case, described by Morel et al., in which the lesion extended from the sigmoid notch to the mandibular midline [7].

In eighteen cases, lipid hematic values were not investigated but, however, patients did not present a clinic suggestive for metabolic pathologies.

In another three papers, a patient presented a silent local history except for one local trauma to the left ear bone in childhood, a case of a recent third molar extraction, a previous simple bone cyst in the area of the xanthoma, and a venous mucous malformation adjacent to the bone xanthoma [8,9,10,11]. Most of the patients were asymptomatic and so a lot of these lesions were incidentally discovered during a routine screening panoramic radiography. Ten patients presented a painless swelling and three a painful regional asymmetry. In the literature, we even found a case of numbness [12].

We did not identify a unique radiological aspect throughout the case reports. Thirteen lesions showed poorly defined changes in the bone texture, while in twenty-eight cases the lesion showed a clear or even sclerotic margin.

As far as the internal structure is concerned, 24 radiolucent, 2 radiopaque, and 16 mixed lesions were described, from unilocular radiolucent aspect to diffuse honeycomb or ground-glass appearance.

Some signs attributable to malignant lesions, such as five cases of root resorptions and cortical bone erosion in six patients, were also described.

Histological findings were dominated by sheets of foamy cells, which are macrophages with central small, round nuclei, and a cytoplasm filled with droplets of fat, thus giving a foamy aspect. Xanthoma cells were spread in a fibrous tissue, sometimes with fibroblast cells without a storiform organization. A partial encapsulation was noted in four cases reported by Daley et al., and, while Rawal et al. specified the absence of a capsule, the other authors described an infiltrative pattern.

In fact, there were intralesional bone trabeculae between the xanthoma cells. In five patients, there were dystrophic calcifications that explained the radiopaque appearance of same lesions.

The majority of cases lacked any significant inflammation, but 18 lesions presented an infiltration of lymphocytes. Three cases showed cholesterol granulomas with giant cells [13].

Intralesional haemorrhages were reported in nine cases [13,14].

Usually, necrosis, nuclear pleomorphism, and mitosis were not present.

In seven cases, only an incisional biopsy was performed; four patients were treated with simple enucleation, twenty-eight with an additional curettage with the preservation of the tooth involved that, on the contrary, were extracted in four cases.

After a variable follow-up period from 6 months to 18 years, one real case of relapse and four cases of a simple increase in the dimension were reported.

## 3. Discussion

Intraosseous xanthoma occurs at any age but is infrequent in the paediatric population. Most documented cases were seen between the third and fifth decades [1,10]. Males are affected twice as much as females [15].

We found only forty-three cases in the literature, but the real incidence of primary xanthomas of the jaw is hard to establish because of the absence of the typical skin lesions, the frequent asymptomatic clinic presentation, and the lack of pathognomonic radiologic features. The difficulty in finding all the lesions reported in the literature is also due to the frequent use of incorrect terminology such as xanthogranuloma, lipogranuloma, or simply histiocytic lesion of the jaw [11].

We encountered the same problem in the use of the terms in the study of vascular pathologies [16,17].

Most of jaw lesions were reported in the second and third decades of life; however, they were seen across a wide age range from 11 to 72 years with a mean age of 29.14, while the maxillary xanthomas seemed to appear at an older age (fifth and sixth decades).

We did not note gender or race predilection. All cases were monostotic lesions and they were located in the mandible more frequent than the maxillary bone, above all posterior mandibles. Interestingly, other bone sites frequently affected by xanthoma are pelvis, rib, skull, ilium, and tibia, which are all composed of compact and trabecular bone, just like the mandible [2,9].

Many of the xanthomas were small at the time of diagnosis, between 1 and 3 cm.

Extragnathic intraosseous xanthoma are mostly painful, while jaw xanthoma is often incidentally discovered during dental investigations [11,14].

Maxillary lesions seemed to be more painful than mandibular ones, and there was only one patient with numbness of lower lip.

The radiographic appearance was variable and nonspecific: it may range widely from a small, well-defined radiolucent lesion with sclerotic margins to a diffuse areas of heterogeneous density with ill-defined borders [7,11,15,18]. The radiographic aspect of the central part of xanthoma varies from a completely radiolucent lesion to a mixed honeycomb or ground-glass disease with calcifications in the osteolytic areas [13,19,20]. Margins can also be highly variable with presentations including corticated, scalloped, ill-defined, and sclerotic [21].

Computed tomography shows loss of normal trabecular pattern in the medullary cavity, and the lesion may have a cystic aspect or a higher density than the normal bone marrow [15].

Primary bone xanthoma does not seem to significantly affect the surrounding anatomical structures but mostly presents a bone expansion; however, sometimes, it may present root resorption and cortical bone erosion mimicking malignant lesions [11,14].

Magnetic resonance imaging has been used and showed mixed hypointensity–hyperintensity on both T1 and T2 weighted MRI [7,8,20], such as in the case reported here.

Scintigraphy was not helpful: the scholars reported an increased uptake in three cases and no uptake of the radionuclide in another two cases [11,15].

The differential diagnosis for patients presenting with lytic lesions is broad and depends on the patient’s age and the presence of other systemic disease [22,23,24].

The rarity of the lesion and the non-specific radiological characteristics make it difficult to diagnose xanthoma by imaging findings, and a biopsy is necessary in our opinion.

In all cases, the xanthoma cells were the dominant cell type, and they were arranged in cohesive or loose sheets spread in an irregular fibrous tissue.

CD68 and HLA-DR positivity identify activated macrophages and S100 and CD1a negativity excluded lesions containing Langerhans cells [12,15].

The nature of primary xanthoma remains unclear, and controversy exists whether the xanthoma is a reactive process or a benign neoplastic process [12].

The pathogenesis consists of lipid leakage from the blood vessels in tissues, with subsequent phagocytosis of this material by the macrophages. Non-degenerated cholesterol accumulates within the cytoplasm, leading to the presence of the typical foamy cells. Cholesterol clefts also induce an inflammatory giant cell reaction resulting in irregular fibrosis.

A common theory suggests that lipid leakage is due to local trauma or haemorrhage and in our revision we found foci of haemorrhage and inflammatory infiltration [1,25].

However, some factors allow us to acknowledge xanthoma as a benign neoplastic process, since it exhibits tumour growth by expansion (non-infiltrative in bone tissue) and the apparent spontaneous occurrence in the absence of trauma, infections, or precipitating systemic disease [12,14].

Only one patient reported a trauma in the left ear bone in childhood and no patient presented had dental disease; conversely, the teeth involved were vital or already devitalized [8].

According to other authors, as a pathogenetic theory, primary xanthoma could be a secondary regressive change in long-standing pre-existing lesions, such as bone cysts, fibrous dysplasia, and aneurismal bone cysts [7]. Only Mosby et al. described the case of a xanthoma arising in a patient with a known simple mandibular cyst in the same site [10]. In support of the regressive theory, Harsany et al. described the formation of more xanthomatous material in repeated biopsy specimens of the same lesion [11].

Another theory suggests xanthomatous transformation of undifferentiated mesenchymal cells by lipotropic factors in the blood in patients with autoimmune conditions [26].

There have been no cases of spontaneous resolution in the literature: all cases were treated with curettage never requiring radical bone resection and only one recurrence has been reported [12,21]. However, it is necessary to consider the large discrepancy in follow up between the various cases, in some very short, and the tendency of the lesion to increase in size in some cases. Moreover, only four lesions presented a partial capsule, all the other exhibits infiltrate the lamellar bone. Therefore, the prognosis seems to be good even with a mild curettage and the saving of noble structures, but careful clinical and imaging follow-up recommendations seems to be indicated for safety [2,14]. However, no guidelines are available as in other ultra-rare diseases [27,28].

Bone graft in residual cavity is possible, but only Yamada et al. performed this in his research [8].

In xanthomas secondary to metabolic disorders, the lesions tend to recur after surgery, even if radiologically and histologically appearance was like primary xanthoma. Therefore, in our opinion, it is important to perform a biochemical analysis on lipid values to prevent a relapse.

The temporomandibular joint involvement is interesting and peculiar in the present case report. In this case, this was more helpful than the possibility to use a conservative treatment with functional preservation.

Our patient had a long follow-up period of six years without any signs of recurrence.

## 4. Suggestions for Future Guidelines

Primary xanthoma of the jaws are often incidental findings in orthopanoramics performed for dental treatment.

Magnetic resonance and scintigraphy are not helpful, and we recommended computed tomography as radiological study.

Moreover, the radiographic appearance is variable, nonspecific, and can mimic a malignant lesion and so, in case of bone lesions, we always suggest an incisional biopsy.

In our case, the radiological characteristics were benign and so we immediately performed an excisional biopsy to avoid a second surgical aggression to condylar process with possible ankylosis.

According to the literature, enucleation and conservative curettage guarantee sufficient control preserving noble structure and teeth. In our case, a conservative treatment allowed us to preserve the temporomandibular joint function.

There is a limited number of articles concerning the surgical treatment of primary xanthoma of jaw and all publications are retrospective case series or simple case report. No prospective studies have been performed to verify the efficacy of simple curettage. Most studies had a short follow-up period, insufficient to verify the long-term stability of the results. For all these reasons, even if no recurrences have been reported in the literature, we think that a clinic and radiological follow up with OPT or, even better, CBCT every 6 months in the first year, and then yearly, is advisable.

## 5. Conclusions

The diagnosis of primary mandibular xanthomas was difficult, mainly because of the absence of typical cutaneous manifestations and alterations in lipid values characteristic of the secondary tumour. The radiographic appearance was variable and nonspecific and so, in our opinion, the diagnosis requires the correlation of clinical and radiographic features with histopathological findings.

We and the literature recommend conservative enucleation with preservation of noble structure, but we even suggest a clinical and radiological follow up.

The pathogenesis of primary xanthomas is poorly understood, as is their predilection for compact and trabecular bone. A lot of work to better understand their behavior still has to be done.

## Figures and Tables

**Table 1:** Demographic data, clinic radiographic, and histological features of the cases retrieved in the literature review.

**Age**	Mean	29
	First decade	0
	Second decade	15
	Third decade	13
	Fourth decade	4
	Fifth decade	6
	Sixth decade	3
	Seventh decade	1
	Eighth decade	1
**Sex**	F	24
	M	19
**Race**	Caucasian	41
	African-American female	2
**Anatomical location**	Mandible - Anterior - Posterior Maxilla - Anterior - Posterior	40 4 36 (22 Left; 14 Right) 3 2 1 Left
**Size**	Size reported	21
	1 cm	4
	1–2 cm	7
	2–3 cm	5
	>3 cm	5
**Local history**	Trauma	1
	Previous bone lesion	1
	Previous mucosal lesion	2
	Unerupted tooth	4
	Recent tooth extraction	1
	Orthodontic therapy	1
**Symptomatology**	Asymptomatic	29
	Painless swelling	10
	Painful swelling	3
	Numbness	1
**Radiological features**	Margins - Well-defined - Ill-defined Inner appearance - Radiolucent - Radiopaque - Mixed Root resorption Cortical resorption	28 13 24 2 16 5 6
**Treatment**	Incisional biopsy	7
	Partial enucleation	2
	Enucleation	2
	Enucleation with curettage	28
	Enucleation and tooth extraction	4
**Histology**	Encapsulated	4
	Foamy cells	43
	Fibrous tissue	43
	Fibroblastic proliferation	3
	Adipocyte	4
	Inflammatory infiltrate	18
	Lamellar bone fragments	8
	Giant cells	3
	Cholesterol cleft	3
	Dystrophic calcification	5
	Haemorrhage	9
**Relapse**	Yes	1
	No	38
	Minimal Enlargement	4

## Data Availability

No new data were created or analyzed in this study. Data sharing is not applicable to this article.

## Supplementary Materials

The following supporting information can be downloaded at: https://www.mdpi.com/article/10.3390/reports6010006/s1, Table S1: Reported cases of primary xanthoma of the jaw.
