# Supplementary material for: Primary Mandibular Condyle Xanthoma: Case Report and Literature Review
Source: Reports (MDPI). 2023 Feb 15;6(1):6. doi: 10.3390/reports6010006 (PMC12225317; doi:10.3390/reports6010006)
Supplement: Supplementary file 1 [file reports-06-00006-s001.zip › reports-2161913-supplementary.pdf]

Table S1. Reported cases of primary xanthoma of the jaw.

Authors	Year	Age	Sex	Site	Size cm	Symptoms	Radiologica features	Comorbidity	Treatment	Relapse	Histology	Follow-up
**Mosby et al.**	1983	28	F	L. mandible	-	-	-	A simple bone cyst 7 years previously<br>Normal hematologic value	Enucleation and curettage	no	Sheet of foamy macrophage in enlarged marrow spaces<br>Scanty fibrovascular network<br>Trabeculae of mature vital bone<br>Inflammatory cells mainly lymphocytes	-
**Harsanyi and Larsson**	1988	16	F	L. mandible	-	Painless swelling<br>? fibrous dysplasia	multilocular from condylar neck to midline, root resorption, buccolingual expansion<br>no uptake of radionuclide	Normal hematologic value	Partial enucleation and third molar extraction	Very slow expansion	Non encapsulated,<br>Sheet of foam cells in fibrous tissue.<br>Inflammatory cells<br>S100-, αantitrypsina, lys	18 y
		23	M	R. mandible	2,3x1,8	-<br>? ossifying fibroma	radiopaque/radiolucent, sclerotic margin	Not available	Only two biopsy	Minimal enlargement	Non encapsulated,<br>Sheet of foam cells in fibrous tissue.	8 y
		27	F	R mandible		Painless swelling<br>Change occlusion<br>? myeloma-lymphoma	multilocular /ill-defined honeycomb,<br>no uptake of radionuclide	Normal hematologic value	Only three biopsy	enlargement	Non encapsulated,<br>Sheet of foam cells in fibrous tissue.<br>Inflammatory cells, S100-, αantitrypsina, lys	8 y
		13	F	R mandible		Painless swelling<br>? fibrous dysplasia	Diffuse ground-glass<br>Buccolingual expansion, incompletely erupted second premolar<br>Intense radionuclide uptake	Not available	Only biopsy	no	Non encapsulated,<br>Sheet of foam cells in fibrous tissue.<br>Inflammatory cells, immature bone	4 y
		12	F	A mandible		Painless swelling	Diffuse honeycomb<br>Radionuclide uptake<br>Angiography ruled out intraosseous hemangioma	Venous mucous malformation<br>Normal hematologic value	Only biopsy<br>Mv completely removed	Slight enlargement	Non encapsulated,<br>Sheet of foam cells in fibrous tissue.<br>Inflammatory cells	2 y
		15	F	L mandible		Painless swelling<br>? cyst	Diffuse ground-glass	Normal hematologic value	Only biopsy	No increasement	Non encapsulated,<br>Sheet of foam cells in fibrous tissue.<br>Inflammatory cells<br>αantitrypsina, lys	2 y
		72	F	L mandible	2,5x1	Painless swelling<br>?osteoma	Bony mass	no	Partial excision	no	Non encapsulated,<br>Sheet of foam cells in fibrous tissue.<br>Inflammatory cells<br>Fibroblast<br>bone lamellae	1 y
**Slootweg et al.**	1993	49	M	L Mandible		Painless swelling	Ill-defined radiopacity with mandibular contour deformation	Not available	curettage		Foam cells in marrow spaces, multinucleated giant cells and cholesterol cleft, old and fresh hemorrhage bone lamellae, calcified material	-
**Mateo et al**	2004	11	M	L mandible		Painless swelling	Ill-defined radiolucent lesion with one unerupted canine<br>Radionuclide hypercapture	unerupted canine<br>Normal hematologic value	Enucleation with tooth preservation	No<br>Correct eruption of the canine	Foam cells and immature fibrous tissue and adipocyte, inflammatory cells<br>CD68+	5 y
**Ramos-Perez et al**	2011	25	M	L mandible	2	-	Ill-defined unilocular radiopaque/radiolucent<br>Adjacent to distal root of the third molar	Local history negative<br>Normal hematologic value	Enucleation and curettage	no	Foam cells, scarce fibrous connective and adipocytes<br>CD68 +	2 y
**Daley et al.**	2015	56	M	A-L mandible	1,1x3.5 cm	Numbness and swelling	Scalloped radiolucency 1,1 x 3.5 cm	Not available	Curettage, with tooth preservation	no	Sheet of foam cells<br>Fibrous tissue, giant cells with cholesterolcleft,<br>Lymphocytes and plasma cells, hemorrhage<br>CD68 +, HLA-DR +<br>S100 -, CD1a -	2y
		24	M	L mandible	2x2 cm	-	Radiolucency 2 x 2 cm corticated	Not available	Curettage, with tooth preservation	no	Partially encapsulated, Sheet of foam cells<br>Fibrous tissue<br>Lymphocytes and plasma cells<br>CD68 +, HLA-DR +<br>S100 -, CD1a -	6 y
		47	M	L mandible	3x2 cm	-	Radiolucency 3 x 2 cm	Not available	Curettage, teeth removed	no	Partially encapsulated, Sheet of foam cells<br>Fibrous tissue<br>Lymphocytes and plasma cells, hemorrhage<br>CD68 +, HLA-DR +<br>S100 -, CD1a -	5,5 y
		48	M	Ant maxilla		Painless swelling	Radiolucency, labial plate perforation	Not available	Curettage, with tooth preservation	no	Partially encapsulated, Sheet of foam cells<br>Fibrous tissue<br>Lymphocytes and plasma cells, hemorrhage<br>CD68 +, HLA-DR +	lost

											S100 -, CD1a -	
		22	M	R mandible		-	Radiolucency	Not available	Curettage, with tooth preservation	no	Partially encapsulated, Sheet of foam cells Fibrous tissue Lymphocytes and plasma cells, hemorrhage CD68 +, HLA-DR + S100 -, CD1a -	1 y
**De Araujo et al**	2015	14	F	L mandible	1 cm	-	Unilocular radiolucency, between the root of premolar teeth without root resorption, 1 cm, irregular margins	Local history negative Norma hematologic value	Curettage, with tooth preservation	No, smaller lesion although present	Foam cells, discrete fibrous stroma CD68 , vimentin +, scarcity of S100 +, scarcity of CD1a +	1 y
**Morel et al**	2015	40	F	L mandible	From the sigmoid notch to the mandibular midline	Painful swelling	Honeycomb-type multilocular hemi-mandibular enlargement, radiolucent-radiopaque CBCT T1-T2 hypointense and hyperintense	Local history negative Norma hematologic value	Bone biopsy	No increase in size	Foam cells, loose fibrous connective tissue, trabecular bone, no inflammatory infiltrate,CD 68 +, CD1a- S100-	6 month
**Rawal et al**	2016	22	F	R mandible		-	Unilocular circumscribed	Not available Local history negative	Aggressive curettage	no	Foam cells, fibrous tissue CD68 +, CD 163 +	12-1 y
		25	M	L mandible		-	Multilocular circumscribed, sclerotic border	Local history negative Norma hematologic value	Aggressive curettage	no	Foam cells, fibrous tissue CD68 +, CD 163 +	12-1 y
		15	M	R mandible		-	Unilocular circumscribed	Local history negative Norma hematologic value	Aggressive curettage	no	Foam cells, fibrous tissue CD68 +, CD 163 +	12-1 y
		12	F	R mandible		-	Unilocular circumscribed	Local history negative Norma hematologic value	Aggressive curettage	no	Foam cells, fibrous tissue CD68 +, CD 163 +	12-1 y
		58	F	A maxilla		Painful swelling	Unilocular circumscribed	Local history negative Norma hematologic value	Aggressive curettage	no	CD68 +, CD 163 +	12-1 y
		49	F	L mandible		-	Unilocular circumscribed	Local history negative Norma hematologic value	Aggressive curettage	no	Foam cells, fibrous tissue CD68 +, CD 163 +	12-1 y
		36	M	R mandible		-	Unilocular circumscribed Root resorption, inferior dental canal displaced, lingual perforation	Local history negative Norma hematologic value	Aggressive curettage	no	Foam cells, fibrous tissue, inflammatory infiltration CD68 +, CD 163 +	12-1 y
		35	M	L mandible		-	Unilocular circumscribed	Local history negative Norma hematologic value	Aggressive curettage	no	Foam cells, fibrous tissue CD68 +, CD 163 +	12-1 y
		53	F	L maxilla		Pain and loose teeth	Unilocular circumscribed, Root resorption	Local history negative Norma hematologic value	Aggressive curettage	no	Foam cells, fibrous tissue CD68 +, CD 163 +	12-1 y
		63	F	L mandible		-	Unilocular circumscribed, Root resorption	Local history negative Norma hematologic value	Aggressive curettage	no	Foam cells, fibrous tissue CD68 +, CD 163 +	12-1 y
**Yamada et al**	2016	27	F	L mandible	1x2 cm	-	Unilocular radiolucent, irregular margins 1x 2 cm. ipointensity T1, iperintendity T2, adjacent to distal root of the lower third molar	History of local trauma Norma hematologic value	Enucleation and third molar extraction and bone graft	no	Foam cells, lymphocytes, fibrous tissue CD68+, AE1/AE3-,S100-	4 y
**Saha et al**	2018	30	M	A mandible		-	Ill-defined lesion from teeth 32 to 43, with buccal cortical plate erosion but no cortical expansion. OPG, CBCT	no	Complete excision	no	Foamy cells, stromal fibrosis, Dystrophic calcification No inflammation	1 y
**Arruda et al**	2019	23	M	PR mandible	2.0	-	Well-defined/sclerotic margin Radiolucent unilocular	Recent third molar extraction	curettage	no	Foamy cells and scarce connective tissue, trabecular bone CD 68+, S100-	3,5 y
		21	F	PR mandible	3.5	-	Ill-defined/punched-out margin Radiolucent unilocular	No available	curettage	no	Foamy cells and scarce connective tissue, trabecular bone CD 68+, S100-	1 y
		45	F	AR mandible	2.5	-	Well defined/punched-out margin Radiolucent unilocular Root resorption and cortical bone resorption	No available	curettage	no	Foamy cells and scarce connective tissue, trabecular bone CD 68+, S100-	2,5 y
		19	F	PR mandible	3.0	-	Well defined/punched-out margin Radiolucent multilocular	Norma hematologic value	curettage	no	Foamy cells and scarce connective tissue, trabecular bone CD 68+, S100-	5 y

		13	F	PL mandible	3.5	-	Ill-defined/punched-out margin<br>Radiopaque/radiolucent multilocular<br>Cortical resorption and expansion	No available	curettage	no	Foamy cells and scarce connective tissue, trabecular bone<br>CD 68+, S100-	1 y
**Wilkinson et al**	2020	28	F	PL mandible	1	-	Multiloculr radiolucency	Not available hematic value<br>Local history negative	curettage	no	Foamy cells, chronic lymphocytic, foci of hemorrhage, dystrophic calcifications, cholesterol clefts, multimucleated giant cells, mature adipocytes<br>CD 68+, CD1a-, S100-	6-60 months
		17	F	PL mandible	-	-	Well-circumscribed, unilocular radiolucency, area of teeth 18-19	Not available hematic value<br>Local history negative	curettage	no	Foamy cells, chronic , lamellar and woven bone fragments, lymphocytic, foci of hemorrhage, dystrophic calcifications, CD 68+, CD1a-, S100-	6-60 months
		27	M	PL mandible	2.0	-	Multilocular radiolucency with honeycomb pattern and sclerotic margins	Not available hematic value<br>Local history negative	Incisional biopsy	no	Foamy cells, chronic lymphocytic, foci of hemorrhage, dystrophic calcifications, CD 68+, CD1a-,	Lost to follow up
		33	M	PR mandible	1.0	-	Multilocular radiolucency with honeycomb pattern	Not available hematic value<br>Local history negative	Enucleation with aggressive curttage	no	Foamy cells, chronic lymphocytic, foci of hemorrhage, fibroblastic proliferation, mature adipocytes, hyaline globules, CD 68+, CD1a-, S100-	6-60 months
		14	F	PR mandible	1.0	-	Multilocular radiolucency with sclerotic margins	Orthodontic therapy<br>Not available hematic value	curettage	no	Foamy cells, lamellar and woven bone fragments, chronic lymphocytic, foci of hemorrhage, fibroblastic proliferation CD 68+, CD1a-, S100-	6-60 months
		12	F	PL mandible	1.5	-	Radiolucency	Not available hematic value<br>Local history negative	curettage	no	Foamy cells, lamellar and woven bone fragments, chronic lymphocytic, hemorrhage, mature adipocytes, CD 68+, CD1a-, S100-	6-60 months
**Kim et al**	2022	17	M	PR mandible	2.4	Painless swelling	Heterogeneous radiolucent multilocular, honeycomb-like structure, well-defined, sclerotic margins, with third molar germ<br>OPT , CBCT	Normal hematic value	Excisional biopsy, and third molar extraction and curettage, curettage with peripheral ostectomy 1 year later<br>Orthognathic surgery 7 month later	Enlargement of lesion after the first surgery,<br><br>No recurrence after second treatment	Sheets of foamy and ne foamy cells<br>CD68+	2 y after second treatment
**Jones et al**	2022	17	M	PL mandible	Body, angle and ramus	-	Irregular shaped, non corticated mixed radiolucent and radiopaque lesion, well-defined multilocular, with germ of the third molar<br><br>OPT, CBCT	Normal hematic value<br>Local history negative	Incisional biopsy and third molar extraction<br><br>Complete enucleation and curettage with superficial neurolysis and second molar extraction	no	Sheet of foamy cells, bone trabeculae, lymphocytic infiltrates, scattered giant cells<br>CD68+, CD 163+ CD1a -	1 y
